# The size and culturability of patient-generated SARS-CoV-2 aerosol

**DOI:** 10.1038/s41370-021-00376-8

**Published:** 2021-08-18

**Authors:** Joshua L. Santarpia, Vicki L. Herrera, Danielle N. Rivera, Shanna Ratnesar-Shumate, St. Patrick Reid, Daniel N. Ackerman, Paul W. Denton, Jacob W. S. Martens, Ying Fang, Nicholas Conoan, Michael V. Callahan, James V. Lawler, David M. Brett-Major, John J. Lowe

**Affiliations:** 1 Department of Pathology and Microbiology, University of Nebraska Medical Center, Omaha, NE USA; 2 Global Center for Health Security, University of Nebraska Medical Center, Omaha, NE USA; 3 National Strategic Research Institute, Omaha, NE USA; 4 Department of Biology, University of Nebraska Omaha, Omaha, NE USA; 5 Department of Pathobiology, University of Illinois at Urbana-Champaign, Urbana, IL USA; 6 Electron Microscopy Core Facility, University of Nebraska Medical Center, Omaha, NE USA; 7 Vaccine & Immunotherapy Center, Massachusetts General Hospital, Boston, MA USA; 8 Department of Internal Medicine, University of Nebraska Medical Center, Omaha, NE USA; 9 Department of Epidemiology, University of Nebraska Medical Center, Omaha, NE USA; 10 Department of Environmental, Agricultural and Occupational Health, University of Nebraska Medical Center, Omaha, NE USA

**Keywords:** SARS-CoV-2, aerosol transmission, viral aerosol, human-generated aerosol

## Abstract

**Background:**

Aerosol transmission of COVID-19 is the subject of ongoing policy debate. Characterizing aerosol produced by people with COVID-19 is critical to understanding the role of aerosols in transmission.

**Objective:**

We investigated the presence of virus in size-fractioned aerosols from six COVID-19 patients admitted into mixed acuity wards in April of 2020.

**Methods:**

Size-fractionated aerosol samples and aerosol size distributions were collected from COVID-19 positive patients. Aerosol samples were analyzed for viral RNA, positive samples were cultured in Vero E6 cells. Serial RT-PCR of cells indicated samples where viral replication was likely occurring. Viral presence was also investigated by western blot and transmission electron microscopy (TEM).

**Results:**

SARS-CoV-2 RNA was detected by rRT-PCR in all samples. Three samples confidently indicated the presence of viral replication, all of which were from collected sub-micron aerosol. Western blot indicated the presence of viral proteins in all but one of these samples, and intact virions were observed by TEM in one sample.

**Significance:**

Observations of viral replication in the culture of submicron aerosol samples provides additional evidence that airborne transmission of COVID-19 is possible. These results support the use of efficient respiratory protection in both healthcare and by the public to limit transmission.

## Introduction

Since first being reported in December of 2019, Severe acute respiratory syndrome coronavirus 2 (SARS-CoV-2) the etiologic agent of coronavirus disease 2019 (COVID-19), has rapidly spread across the globe. Implementing optimal infection prevention and control (IPC) practices to mitigate disease spread depends upon a robust understanding of the mechanisms of transmission and relative risk of environmental and personal exposures. SARS-CoV-2 was originally considered to spread primarily by droplet and direct contact [1–3]. Similar to other respiratory viruses, including the closely related severe acute respiratory syndrome (SARS-CoV-1) and Middle East respiratory syndrome, the virus can also be transmitted by aerosolized particles [4], mounting evidence supporting the importance of airborne transmission [5–8] has resulted in changes in CDC guidance affirming the importance of aerosol transmission of COVID-19 [9].

In order to classify an infectious disease as airborne, studies must show transmission via aerosol particles. The diameter of particles involved in airborne transmission has been actively debated over the course of the pandemic. Initially, historical guidance utilizing a 5 µm cut-off for airborne transmission was used, but recent work [10, 11], argues that the scientific basis for such a cut-off is non-existent, and that a broader range of aerosol size should be considered to be involved in airborne transmission of disease. Therefore, there are several lines of evidence that must be demonstrated in order to establish airborne transmission: infectious aerosol that is small enough to be transported to and inhaled by another person must be produced by ill individuals; the infectious aerosol must be stable long enough to expose another person; and if inhaled, the viral aerosol must be capable of causing infection. Experimentally, viral growth in medium from expired aerosol particles <5 µm has been detected from influenza infected humans [12, 13] and several studies have also demonstrated culture of SARS-CoV-2 aerosol collected near infected indivuals [14, 15], thus demonstrating the infectious nature of the expired particles. SARS-CoV-2 generated aerosols have been shown to retain infectivity for several hours in the absence of sunlight [16, 17], and extensive contamination of COVID-19 patient care areas with SARS-CoV-2 genomic RNA suggest that aerosol dissemination of virus may occur [18–20]. In addition, recent clusters of COVID-19 cases linked to a church choir practice in Washington, U.S.A. and a restaurant in Guangzhou, China are suggestive of airborne transmission [21, 22]. Finally, the primary receptor of SARS-CoV-2 for infection is understood to be ACE2 [23], which is expressed throughout the human respiratory tract, indicating that inhalation would be a compatible route of infection.

In April 1, 2020, letter to the White House Office of Science and Technology Policy, the National Academy of Medicine Standing Committee on Emerging Infectious Diseases, and 21st Century Health Threats recommended further research into the role of infectious aerosols in the spread of SARS-CoV-2 [24]. The work presented here demonstrates that aerosols containing SARS-CoV-2 RNA exist in particle modes that are produced during respiration, vocalization, and coughing; and that RNA-containing aerosols <5 µm in diameter contain infectious SARS-CoV-2 virions. The infectious nature of aerosol collected in this study, taken with the other lines of evidence presented, illustrates that airborne transmission of COVID-19 is possible, and that infectious aerosol may be produced without coughing.

## Methods

### Sample collection

Aerosol sampling was conducted around six patients in five rooms in two wards (referred to as Ward 5 and Ward 7) on three separate days in April of 2020 (Table S1). Ward 7 was operated with individual rooms at negative pressure to the hallway, while rooms in Ward 5 could not be individually isolated. In the case of Ward 5, the entire ward was put into negative airflow with the rest of the floor. In Ward 7, ventilation, heating and cooling are controlled centrally. In Ward 5, each rooms has central ventilation and there is a local fan coil unit in each room for heating and cooling. Room surfaces were disinfected daily. An Aerodynamic Particle Sizer Spectrometer (APS 3321; TSI, Inc., Shoreview, MN) was used to measure aerosol concentrations and size distributions from 0.5 µm up to 20 µm. A NIOSH BC251 sampler [25, 26] was used to collect size-segregated aerosol samples for both rRT-PCR and culture analysis. The BC251 is a dry air sampler that when operated at 3.5 Lpm provides three stages of size-segregated aerosol, >4.1 µm particles collected onto a 15 mL conical tube, 1–4 µm collected onto a 1.5 mL conical tube, and a 37 mm filter that collects particles <1 µm. In these studies, a gelatin filter (Sartorius, GmbH) was used in the final stage for preservation of viral integrity [27]. During these studies, APS measurements were taken at 1 min intervals for 30 min in each room, with the exception of Room 5C where loss of battery power to the laptop collecting the data aborted sampling after 10 min. A single BC251 sampler was run concurrently, and collocated, with the APS for 30 min in each room at the foot of each patient’s bed with both inlets at the same height, ~1.1 m from the floor. A blank 37 mm gelatin filter was used as a control for each day to monitor for any cross-contamination of samples during handling and processing.

### Sample recovery, RNA extraction and real-time reverse transcriptase PCR

Recovery of the samples collected using the BC251 aerosol sampler was performed by addition of 5 mL of sterile phosphate buffered saline (PBS) to the 15 mL conical tube, 1 mL of sterile PBS to the 1.5 mL conical tube, and by placing the 37 mm gelatin filter from the last stage in a 50 mL conical tube and dissolving into 10 mL of sterile PBS. Samples were recovered within 1–2 h of collection. RNA was isolated from recovered samples immediately after processing.

To recover viral RNA, 400 uL of each recovered sample was processed using a Qiagen DSP Virus Spin Kit (QIAGEN GMbH, Hilden, Germany). A negative extraction control (no sample added) was included with each set of extractions. Samples were eluted in 50 uL of Qiagen AVE Buffer. rRT-PCR was performed using Invitrogen Superscript III Platinum One-Step Quantitative rRT-PCR System. Each rRT-PCR run included a positive synthetic DNA control and a negative, no template, control of nuclease free water. Reactions were set up and run with initial conditions of 10 min at 55 °C and 4 min at 94 °C then 45 cycles of 94 °C for 15 s and 58 °C for 30 s, on a QuantStudio^™^ 3 (Applied Biosytems^™^, Inc) with the reaction mix and primers and probe targeting the E gene of SARS-CoV-2 [20, 28] (details provided in Table S2). Each sample was run in triplicate.

To quantify the virus present in each sample from the measured Ct values obtained from the rRT-PCR, a standard curve was developed using RNA extracted from a known quantity of SARS-CoV-2 virus (BEI_ USA-WA1/2020) cultivated in Vero E6 cells (using the same method described below for environmental samples). A five-log standard curve was run in triplicate beginning at a concentration of 1 × 10^2^ pfu/mL, as determined by plaque assay. The data were fit with the exponential function:

1 $${{{{{\mathrm{Equivalent}}}}}}\,{{{{{\mathrm{Viral}}}}}}\,{{{{{\mathrm{Titer}}}}}}\left( {\frac{{pfu}}{{mL}}} \right) = 6.0E9 \ast e^{ - 0.707 \ast C_t}$$

In the equation, *C*_*t*_ is the cycle time where amplification is definitively above the background. The same cycle threshold was used for all rRT-PCR runs in this study. Equation 1 was used to convert measured *C*_*t*_ values to an equivalent viral titer (e-pfu) for each sample. This calibration with extracted viral RNA more accurately represents the RNA that can be recovered from virus through the extraction and reverse-transcriptase processes, which are not accurately captured using a synthetic DNA control. However, e-pfu may not accurately represent the number of copies associated with a virion in an environmental sample, as compared to one grown in cell culture. Therefore, the number of RNA copies/mL as a function of viral titer (pfu/mL) was determined for this culture. A synthetic DNA control for the SARS-CoV-2 E gene (Integrated DNA Technologies, Iowa, USA) that had an initial concentration of 2 × 10^8^ copies/mL was diluted and assayed via qPCR using identical assay conditions as RNA extracted from the viral culture (see above). The number of copies per pfu from cell culture was compared over five orders of magnitude in concentration, with *C*_*t*_ values from 23 to 38, and the average was calculated to be 1.35 × 10^6^ ± 6.29 × 10^5^ RNA copies/pfu. This was then used to determine the RNA copies/L of air in collected samples.

### Cell culture and detection of viral replication

Vero E6 cells were used to culture SARS-CoV-2 virus from environmental samples within 1–3 days following collection. The cells were cultured in Dulbeccos’s minimal essential medium (DMEM) supplemented with heat inactivated fetal bovine serum (10%), penicillin/streptomycin (10,000 IU/mL & 10,000 µg/mL) and amphotericin B (25 µg/mL). For propagation of infectious virus from the aerosol samples, 100 µL of undiluted samples, and a mock infection of 100 µL of SARS-CoV-2 RNA extracted from a culture at 1 × 10^2^ TCID_50_/mL, were added to 24-well plates containing confluent monolayers of Vero E6 cells and 3 mL of supplemented DMEM (described above) and grown at 37̊ °C with 5% CO_2_. In addition, three additional infection control experiments were performed using laboratory stock of SARS-CoV-2. In these experiments, the supplemented DMEM was inoculated with stock virus to reach supernatant concentrations of 1.6, 1.6 × 10^–1^, and 1.6 × 10^−2^ pfu/mL. To look for evidence of viral replication, RNA was extracted from 140 uL of supernatant from days 1 and either day 5 or 6 of incubation, using the QIAamp Viral RNA Mini Kit (QIAGEN GMbH, Hilden, Germany) and rRT-PCR was performed on these samples following the methods described for environmental samples. The percent increase from day 1 to day 5 or 6 was calculated for all samples to look for evidence of viral replication in each sample. Definitive replication was considered to occur for rRT-PCR samples in which a significant increase in RNA was detected in the supernatant (student’s *t* test, Microsoft Excel, *P* < 0.05). After 6 days, all cell supernatants and lysates were harvested. For samples with statistically significant evidence of replication, subsequent examination of the samples was performed by electron microscopy and western blot assay. Western blots were generated by harvesting cell lysates in RIPA lysis buffer. Twenty microliters of lysate was combined with a 2× reducing sample buffer (Invitrogen), boiled and subjected to sodium dodecyl sulfate polyacrylamide gel electrophoresis analysis followed by Western blot using the antibody against SARS-CoV N protein and glyceraldehyde 3-phosphate dehydrogenase (GAPDH) (Abcam). The gel was examined for protein bands between 40 and 55 kDa, consistent with the size of the SARS-CoV-2 N protein. For electron microscopy, samples were fixed, processed, sectioned, and inspected (see Table S3) on a Tecnai G2 Spirit TWIN (Thermo Fisher Scientific) at 80 kV.

### Aerodynamic data analysis

APS measurements were taken at 1 min intervals and exported as mass concentration in *dM/dlogD*_*p*_ per size bin, assuming particles of unit density. The first bin of the APS data files (<0.523 μm) was discarded during data analysis. An average concentration across all bins for all 1 min measurements was calculated. The averaged raw distributions were then parameterized into two log-normally distributed aerosol modes. The parameterized distributions were assumed to be lognormal and the Solver function in Microsoft Excel was used to calculate the fit parameters, *M*, the total mass concentration, *d*_*pg*_, the mass median aerodynamic diameter, and *σ*_*g*_ the geometric standard deviation for each mode by using a minimization of the sum of least square errors between the raw distribution and parameterized distributions [29]. The total mass of each mode contributing to the raw distributions was summed from 0.542 to 0.97 μm, 1.04 to 3.79 μm, and 4.07 to 9.85 μm to reflect the size ranges overlapping with the different stages of BC251 aerosol sampler. The total concentration in each size bin was normalized such that both the APS parameterized modes and modes obtained from the rRT-PCR analysis performed off the BC251 samples could be compared directly.

## Results

**Fig. 1:**
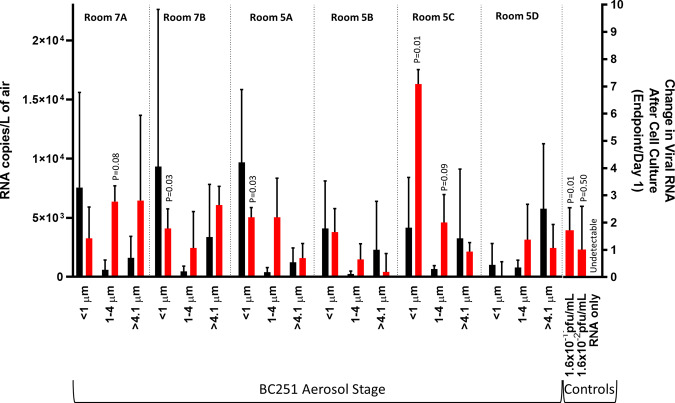
Measured Airborne RNA Concentrations and Associated Percent Change in Viral Copies in Cell Culture. Measured rRT-PCR RNA copies/L of air (black) indicate in initial viral RNA concentrations while the change viral RNA (red) indicates viral replication in cell culture (or lack thereof). Error bars indicate the standard deviation of the RT-PCR-derived concentrations (for air concentration) and calculated measurement uncertainty (for percent change in cell culture). The *P* value comparing the RNA in cell culture supernatant on day 1 vs RNA on the final day is shown if the change is positive and the *P* value is <0.1. Three of the six submicron filter samples indicated an increase in cell culture that was significant (*p* < 0.05) based on the Students *T* Test (7B, 5A and 5C. Two of the 1–4 um samples had *P* values < 0.1, but not <0.05 (7A and 5C). None of >4.1 um samples demonstrated statistically significant replication. Samples with a ratio of <1 indicate a loss of RNA between the first and last day of culture. Cell culture controls (right hand side) indicate the results when known concentration of laboratory cultured virus are introduced, as well as extracted RNA.

In all six rooms, rRT-PCR indicated the presence of viral RNA in all three stages of the BC251 sampler (Fig. 1; Table S4). Furthermore, exposure of recovered samples to Vero E6 cells demonstrated statistically significant viral growth (95% confidence based on *P* < 0.05) after 6 days (5 days in the case of the submicron sample from room 5A) in 3 of the 18 samples (7B, 5A and 5C; Fig. 1). Two of the 1–4 µm samples demonstrated viral growth, between 90 and 95% confidence (7B and 5C; Fig. 1). A single sample had definitive loss of viral RNA during cell culture (5B > 4.1 µm; Fig. 1), as demonstrated by a growth ratio of <1. The mock infection of extracted RNA was undetectable by rRT-PCR after 1 day in cell culture, indicating that amplification observed in cultured aerosol samples was not likely to be from free RNA. The cultures inoculated with known virus showed statistically significant replication at 10^−1^ pfu/mL (shown in Fig. 1) and 10^0^ pfu/mL (shown in Table S4), and no replication at the lowest concentration (10^−2^).

**Fig. 2:**
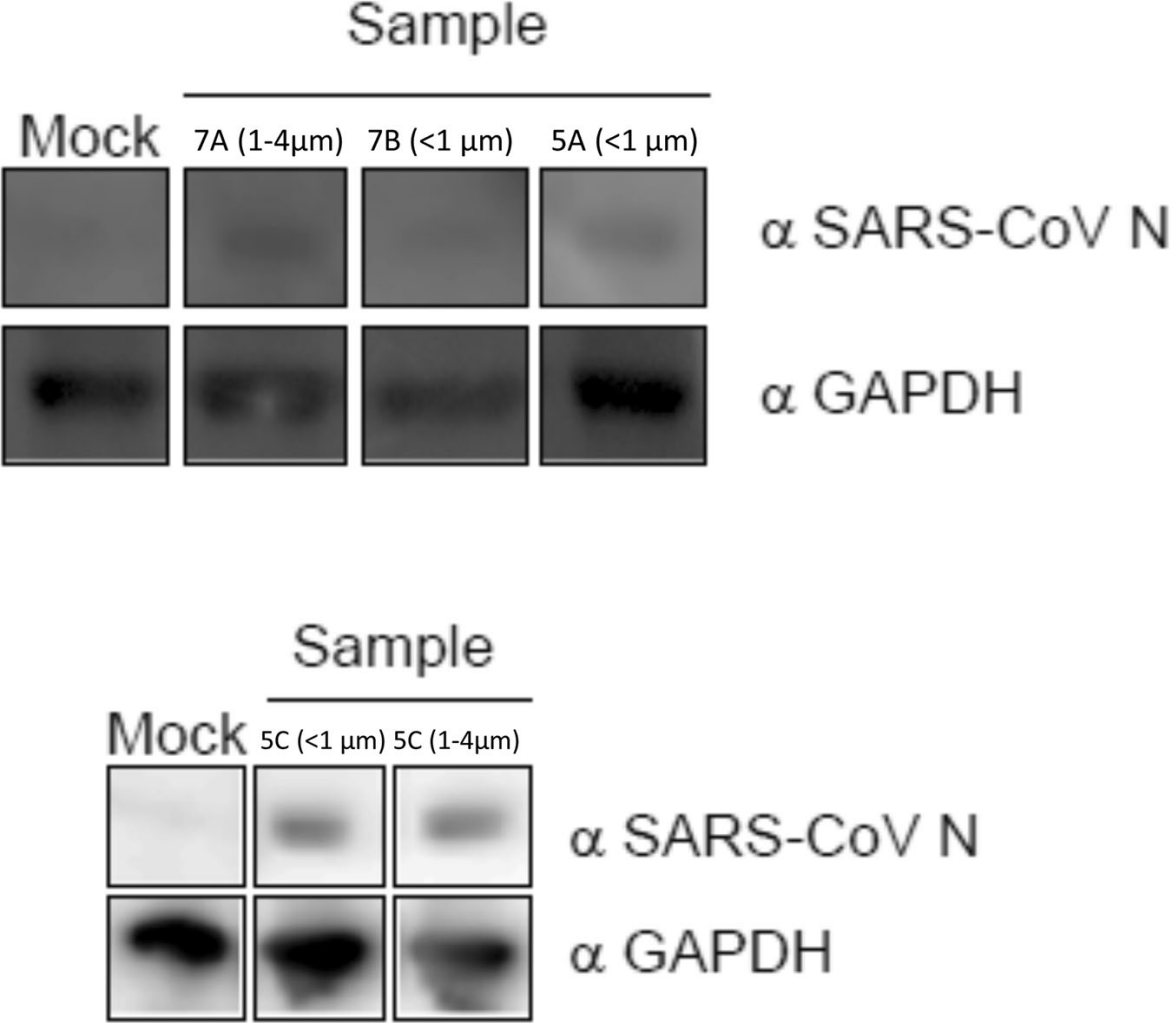
Protein expression of SARS-CoV-2. Cell protein lysates were prepared from the indicated cultured samples and subsequently probed by western blot with a mouse monoclonal anti-SARS nucleocapsid protein (SARS-CoV N) antibody and an anti-GAPDH loading control antibody. The gel images between 40 and 55 kDa are shown. Images of control gels from infections initiated at titers from 10^−2^ to 10^2^ pfu/mL are shown in supplementary Fig. S1, for comparison.

**Fig. 3:**
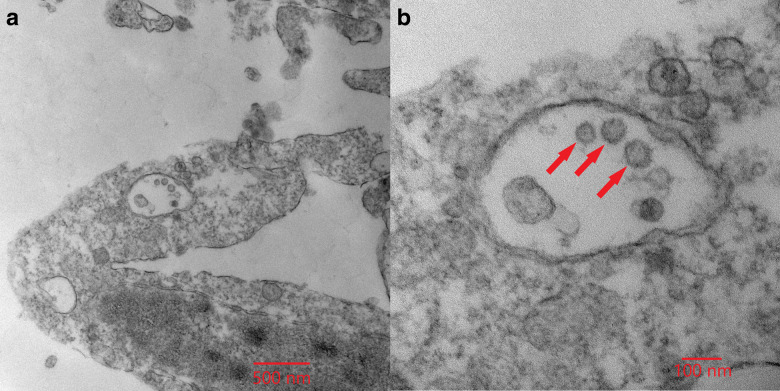
Electron micrographs of SARS-CoV-2 virions cultivated from the sub-micron filter from Room 5C. The same image is shown at two magnifications: (**A**) ×30,000 and (**B**) ×110,000. Identifiable SARS-CoV-2 virions can be seen at both magnifications, and are indicated by red arrows in (**B**).

Cells from the five environmental samples, where replication was indicated at >90% confidence by rRT-PCR, were subsequently examined via western blot, for the presence of viral protein production, and transmission electron microscopy (TEM) for the presence of intact SARS-CoV-2 virions. The presence of SARS-CoV-2 was observed via western blot for all but one of these samples (<1 μm, Room 7B; Fig. 2). Images of control gels (Fig. S1) indicate similar protein bands when infections are started with titers of at least 10^−1^. Intact virus was observed via TEM in the submicron sample from Room 5C (Fig. 3), although it cannot be determined if these virions were produced during cell culture.

The aerosol size distributions measured in the rooms from ward 5 are all similar, while the rooms in 7 have much lower concentrations and broader size distributions than those in ward 5 (Fig. S2). The similarities between rooms in each ward, and the differences between the two wards are likely due in large part to the background aerosol, rather than differences in aerosol produced by the patients. The measured distributions were parameterized, using a log-normal fit and compared with RNA concentrations measured in the BC251, in an attempt to identify a human-generated, viral aerosol mode separate from the background. Two log-normal modes were calculated from the measured APS aerosol mass distributions (Fig. S2 and Table S5). The mean diameter of the small mode distributions is between 0.64 µm and 0.80 µm for all the rooms sampled (Table S5), while the width (GSD) of the distribution varied between 1.17 and 1.30. The large mode distributions had more variability both in terms of mean diameter and width (Table S5). Unfortunately, a statistically defensible relationship cannot be made between any of the APS modes (alone or combined) and RNA extracted from size-segregated aerosol collected by the BC251 (Table S6). Therefore, while patient-generated aerosol is a portion of the measured size distributions, it cannot be separated from the background aerosol in these rooms. Without a more extensive characterization of background aerosol in the each of the spaces, it would likely be impossible to identify the human-generated portion of the aerosol based solely on size distributions.

## Discussion

This study sought to characterize the presence of SARS-CoV-2 in particles consistent with the potential to result in aerosol transmission between patients. Although the aerosol generated by the patient cannot be separated from the background using the size distributions alone, the SARS-CoV-2 RNA extracted from the BC251 stages provides information about which human respiratory activities are responsible for producing those particles. The aerosol collected in the smallest stage of the BC251 (<1 µm) is consistent with particles found in the smallest mode of exhaled breath measured in previous studies [30–33]. This mode of aerosol was observed in all manner of human respiration including breathing, vocalization, and coughing [25] and has been attributed to particles produced in both the bronchial region [33], referred to as the bronchiolar fluid film burst mechanisms [31], and in the larynx [33] The human-associated fraction of the aerosol collected in the 1–4 µm stage of the BC251 are also consistent with production in both the bronchial and laryngeal regions [33], that are produced in greater numbers during vocalization and coughing. The aerosol collected in the largest size bin of the BC251 (>4.1 µm) is almost exclusively composed of those particles in the largest parameterized size mode and likely other particles and droplets not measured by the APS. The human-generated fraction of these larger particles may come dominantly from the oral cavity and laryngeal region with limited to no contribution from the bronchial region [33]. Although viral RNA was consistently observed in aerosol in this largest stage, no evidence of replicating virus was observed in those samples. This finding is consistent with other recent observations by Lednicky [15]. The differences in potential sources of these particles may indicate that particles generated deeper in the respiratory tract are more likely to be culturable.

Observation of fine mode aerosol particles containing infectious SARS-CoV-2 particles leads to several general observations about the potential transmission of SARS-CoV-2. The results of this study, along with the evidence of the stability of SARS-CoV-2 in aerosol [16, 17] and that SARS-CoV-2 infects respiratory tissue [23] provide indications that SARS-CoV-2 may be transmitted via the airborne route. Furthermore, fine mode aerosols are generally produced in the bronchi and the larynx across a wide range of respiratory activities, which is in agreement with previous studies in which SARS-CoV-2 aerosol are observed in the absence of coughing and other symptoms [20] as well as in reports of disease transmission by asymptomatic individuals [21, 22].

The most confident evidence of SARS-CoV-2 replication was detected in the submicron aerosol collected in the last stage of the BC251 sampler. This sampler was not originally intended to collect and preserve the integrity of SARS-CoV-2 in aerosol, and there have not yet been studies to determine how effective this approach might be at preserving the infectivity of the virus. In the first two stages of the sampler, the virus is sampled into two dry conical tubes using inertial impaction. It is possible that this collection mechanism may be degrading the integrity of the virus collected in the 1–4 µm and the >4.1 µm stages. For this study, we employed gelatin filter in the final stage of the BC251 to help preserve intact virus during collection and notably it was in this stage that viral replication was observed. However, Ratnesar-Shumate et al. [34] indicate that gelatin filters and PTFE filters perform similarly and similar to the BC251, as a whole, suggesting that the collection mechanisms used in this study may not have a significant impact on viral stability. These laboratory generated aerosol have different composition and size than human-generated particles, so there may be some limitations in the direct application of these results to the sampling of human-generated aerosol. Therefore, it can be said that infectious SARS-CoV-2 exists in particles <1 µm and may exist in particles up to 4 µm, but it cannot be said that they do not exist in particles larger than four microns, due to the potential for viral decay during aerosol collection.

The large number of gene copies per pfu observed in the rRT-PCR controls may also offer some insight into the challenges in isolating this virus from aerosol samples. The high gene copy to pfu ratio may indicate a large number of defective interfering (DI) particles exist under culture conditions. Other betacoronaviruses are known to produce DI particles in culture. [35] In addition, a similar result has been observed in patient samples. Scola et al. [36] demonstrated that while high viral loads can be measured in patients by rRT-PCR, no culture positive samples were found with Ct values higher than 34. It is unclear if DI particles are involved in these instances, but it is clear that large quantities of RNA are present in both cell culture and human infections even in the absence of high titers of infectious virions. Therefore, despite the ubiquitous RNA contamination observed in the patient environment, it seems that the portion of that contamination that represents infectious virus may be small by comparison. This needs to be considered when interpreting observations of viral RNA in these and other aerosol samples.

## Conclusion

Our results demonstrate that SARS-CoV-2 RNA exists in respired aerosols <5 µm in diameter; that aerosols containing SARS-CoV-2 RNA exist in particle modes that have been demonstrated to be produced during respiration, vocalization, and coughing [33]; and that some fraction of the RNA-containing aerosols contain intact, replication-competent virions (Table S7).

This study supports the role fine mode aerosols in the transmission of SARS-CoV-2, and emphasizes the importance of efficient respiratory protection and airborne isolation precautions to protect from exposure to fine SARS-CoV-2 aerosol when interacting with infected individuals, regardless of symptoms or medical procedure being performed.

Given the ongoing circulation of COVID-19, and recent work highlighting the relative importance of airborne transmission of COVID-19 [5], it is crucial that evidenced-based IPC practices are promoted and implemented to limit the transmission of SARS-CoV-2 in healthcare, and that efficient respiratory protection and hygiene practices are used in public and industry settings. The results of this study, taken with the other lines of evidence presented, further suggests that airborne transmission of COVID-19 is likely in many situations, and that aerosol prevention measures should be implemented to effectively stem the spread of SARS-CoV-2, particularly in crowded settings.

## Supplementary Information

Supplemental Tables and Figures

## Data Availability

All data are available in the main text or the supplementary materials.
