# Supplementary material for: The size and culturability of patient-generated SARS-CoV-2 aerosol
Source: J Expo Sci Environ Epidemiol. 2021 Aug 18;32(5):706–11. doi: 10.1038/s41370-021-00376-8 (PMC8372686; doi:10.1038/s41370-021-00376-8)
Supplement: Supplementary file 1 — Supplemental Tables and Figures [file 41370_2021_376_MOESM1_ESM.pdf]

Supplemental Tables and Figures

Room	Days between admission and sampling	Days between first COVID-19 positive and sampling	Days between most recent COVID-19 positive and sampling	Observations of Respiratory Activities During Sampling	Notes
Room 7A	24	24	1	Breathing, Speaking and Coughing	
Room 7B	7	>10	0 (same day)	Breathing, Speaking and Coughing	transfer from other facility
Room 5A	3	3	3	Breathing, Speaking and Coughing	
Room 5B	2	2	2	Breathing, Speaking and Coughing	
Room 5C	4	3,2	3,2	Breathing and Coughing	2 patients in this room
Room 5D	6	6	6	Breathing only	

Table S1. Metadata for rooms sampled in this study.

**PCR Reaction Mix**	
Nuclease Free Water	6.1 µL
Invitrogen 2X Master Mix	12.5 µL
MgSO4 (50mM)	0.4 µl
Primer/Probe Mix (IDT)* (Primers 10uM, Probe 5uM)	0.5 µl
SuperScript III Platinum Taq	0.5 µl
extracted sample RNA, nuclease free water or positive control	5.0 µL
***E gene target primers and probe:**	
5’/56-FAM/ACACTAAGCC/ZEN/ATCCTTACTGCGCTTCG/3AIBkFG/-3’	Probe
5’-ATATTGCAGCAGTACGCACACA-3'	Primer 1
5’-ACAGGTACGTTAATAGTTAATAGCGT-3'	Primer 2

Table S2. PCR Reaction Mix used for all PCR of SARS-CoV-2 throughout the study.

Process	Reagents	Conditions
Fixing	2% paraformaldehyde in 0.1M Sorenson’s phosphate buffer (pH 7.2)	24 hours at 4 ℃
Washing	0.1M Sorenson’s phosphate buffer (pH 7.2)	3 times
Post Fix	1% aqueous solution of osmium tetroxide	30 minutes
Dehydration series	50,70, 90, 95 and 100% ethanol	
Embedding	50:50 Embed 812 Resin and propylene oxide	Overnight
Final Embedding	Fresh Embed 812 Resin	2 hours at room temperature followed by polymerization at 65 ℃ for 24 hours
Sectioning	100 nm sections stained with 2% Uranyl Acetate followed by Reynolds Lead Citrate	Cut with Leica UC6 Ultracut ultramicrotome

Table S3. Sample preparation for transmission electron microscopy.

Room	Sample Type	Airborne Viral RNA copies/L of Air	Standard Deviation	Day 1 epfu/mL	Day 1 Standard Deviation	Final Day e-pfu/mL	Final Day Standard Deviation	Ratio of Increase in viral RNA	Standard Deviation	Significance of Calculated Increase (P-value)
Room 7A	BC251 <1 µm	7.56E+03	8.04E+03	6.53E-02	4.94E-02	9.24E-02	6.63E-02	1.42	1.15	0.27
Room 7A	BC251 1-4 µm	6.01E+02	8.22E+02	2.75E-02	2.54E-03	7.62E-02	4.32E-02	2.77	0.58	0.09
Room 7A	BC251 4.1 µm	1.61E+03	1.82E+03	4.21E-02	2.33E-02	1.18E-01	1.31E-01	2.81	3.13	0.23
Room 7B	BC251 <1 µm	9.32E+03	1.33E+04	6.47E-02	3.20E-02	1.15E-01	6.09E-02	1.78	0.72	0.05
Room 7B	BC251 1-4 µm	4.56E+02	4.49E+02	8.45E-02	1.10E-01	8.93E-02	3.06E-02	1.06	1.34	0.47
Room 7B	BC251 4.1 µm	3.36E+03	4.47E+03	3.39E-02	7.80E-03	8.95E-02	5.78E-02	2.64	0.69	0.14
Room 5A	BC251 <1 µm	9.69E+03	6.16E+03	3.85E-02	1.02E-02	8.43E-02	2.06E-02	2.19	0.36	0.04
Room 5A	BC251 1-4 µm	3.86E+02	4.02E+02	2.25E-02	2.71E-02	4.93E-02	3.90E-02	2.19	1.44	0.22
Room 5A	BC251 4.1 µm	1.23E+03	1.22E+03	6.57E-02	7.40E-03	4.50E-02	2.39E-02	0.69	0.54	NA
Room 5B	BC251 <1 µm	4.09E+03	4.02E+03	1.08E-01	8.60E-02	1.79E-01	5.77E-02	1.65	0.86	0.14
Room 5B	BC251 1-4 µm	2.37E+02	2.49E+02	7.34E-02	1.37E-02	4.69E-02	2.57E-02	0.64	0.58	NA
Room 5B	BC251 4.1 µm	2.29E+03	4.10E+03	5.53E-01	8.34E-02	9.70E-02	6.41E-02	0.18	0.68	NA
Room 5C	BC251 <1 µm	4.16E+03	4.25E+03	5.37E-02	2.75E-02	3.81E-01	5.61E-02	7.09	0.53	0.01
Room 5C	BC251 1-4 µm	6.50E+02	3.05E+02	5.00E-02	4.68E-02	1.00E-01	4.36E-02	2.00	1.03	0.09
Room 5C	BC251 4.1 µm	3.26E+03	5.85E+03	7.04E-02	1.62E-02	6.53E-02	1.57E-02	0.93	0.33	NA
Room 5D	BC251 <1 µm	1.01E+03	1.81E+03	2.79E-02	1.56E-02	0.00E+00	0.00E+00	0.00	0.56	NA
Room 5D	BC251 1-4 µm	8.02E+02	6.17E+02	5.90E-02	4.87E-02	8.07E-02	8.10E-02	1.37	1.30	0.38
Room 5D	BC251 4.1 µm	5.77E+03	5.49E+03	9.03E-02	2.65E-02	9.54E-02	7.77E-02	1.06	0.87	0.47
Controls										
RNA only				0.00E+00	0.00E+00	0.00E+00	0.00E+00	0.00	0.00	NA
1.6 pfu/mL				6.12E+02	1.04E+02	1.20E+05	6.35E+03	196.08	34.86	0.00
$1.6x10^{-1}$ pfu/mL				2.38E-02	6.15E-03	4.11E-02	1.66E-02	1.72	0.83	0.01
$1.6x10^{-2}$ pfu/mL				2.55E-02	2.70E-02	2.57E-02	3.05E-02	1.01	1.60	0.50

Table S4. Complete data set from Figure 1, indicating viral RNA copy number derived from RT-PCR of raw samples, rRT-PCR-derived viral concentrations from cell culture experiments, and ratio of increase in viral RNA with the statistical significance, (p) as determined by a one-tailed Student's t-test between day 1 and final day rRT-PCR viral RNA concentration.

Room	Small Mode			Large Mode		
	M	σ_g	d_{pg}	M	σ_g	d_{pg}
Room 5A	3.5E-04	1.3E+00	6.4E-01	4.4E-03	1.9E+00	4.2E+00
Room 5B	3.7E-04	1.3E+00	6.8E-01	4.5E-03	1.7E+00	3.8E+00
Room 5C	3.9E-04	1.2E+00	6.7E-01	6.8E-03	2.3E+00	4.3E+00
Room 5D	2.4E-04	1.2E+00	6.7E-01	3.1E-03	3.0E+00	3.9E+00
Room 7A	2.7E-05	1.3E+00	6.9E-01	2.1E-03	2.5E+00	1.1E+01
Room 7B	2.9E-04	1.3E+00	8.0E-01	5.8E-03	1.6E+00	2.3E+00

Table S5. Log-normal parameters calculated for the large and small modes shown in Figure 4.

	Location	SARS-CoV-2 RNA (copies/L)	Measured (µg/L)	Small Mode (µg/L)	Large Mode (µg/L)
<1 µm	Room 5A	9.69E+03	7.95E+00	4.79E+00	3.11E+00
	Room 5B	4.09E+03	6.28E+00	3.05E+00	3.17E+00
	Room 5C	4.16E+03	4.33E+00	3.63E+00	6.45E-01
	Room 5D	1.01E+03	4.61E+00	4.09E+00	5.00E-01
	Room 7A	7.56E+03	4.00E-01	3.05E-01	1.34E-01
	Room 7B	9.32E+03	6.90E+00	3.16E+00	3.45E+00
1 - 4 µm	Room 5A	3.86E+02	4.36E+01	8.50E-02	3.96E+01
	Room 5B	2.37E+02	1.90E+01	1.53E-02	1.68E+01
	Room 5C	6.50E+02	2.88E+01	2.19E-01	2.71E+01
	Room 5D	8.02E+02	3.76E+01	2.65E-01	3.54E+01
	Room 7A	6.01E+02	5.25E+00	2.30E-02	4.51E+00
	Room 7B	4.56E+02	3.76E+01	2.65E-01	3.54E+01
>4.1 µm	Room 5A	1.23E+03	4.59E+01	1.03E-20	3.72E+01
	Room 5B	2.29E+03	1.58E+01	1.39E-29	1.29E+01
	Room 5C	3.26E+03	3.03E+01	9.44E-12	2.86E+01
	Room 5D	5.77E+03	3.13E+01	6.93E-13	2.80E+01
	Room 7A	1.61E+03	1.29E+01	9.29E-14	1.08E+01
	Room 7B	3.36E+03	3.13E+01	6.93E-13	2.80E+01

Table S6. RT-PCR derived virus concentration and recovered mass concentrations for each stage of the BC251 sampler in each room included in this study.

SARS-CoV-2 Detected in Aerosol by PCR and Cell Culture		
	rRT-PCR	Cell Culture
>4.1 µm	6:6 (100%)	0:6 (0%)
1-4 µm	6:6 (100%)	2:6 (33%)*
< 1 µm	6:6 (100%)	3:6 (50%)

Table S7. Number and percentage of aerosol samples where SARS-CoV-2 was detected by rRT-PCR and where replication was observed in cell culture. Samples from the 1-4 µm stage did reach the level of statistical significance ($0.05<P<0.10$).

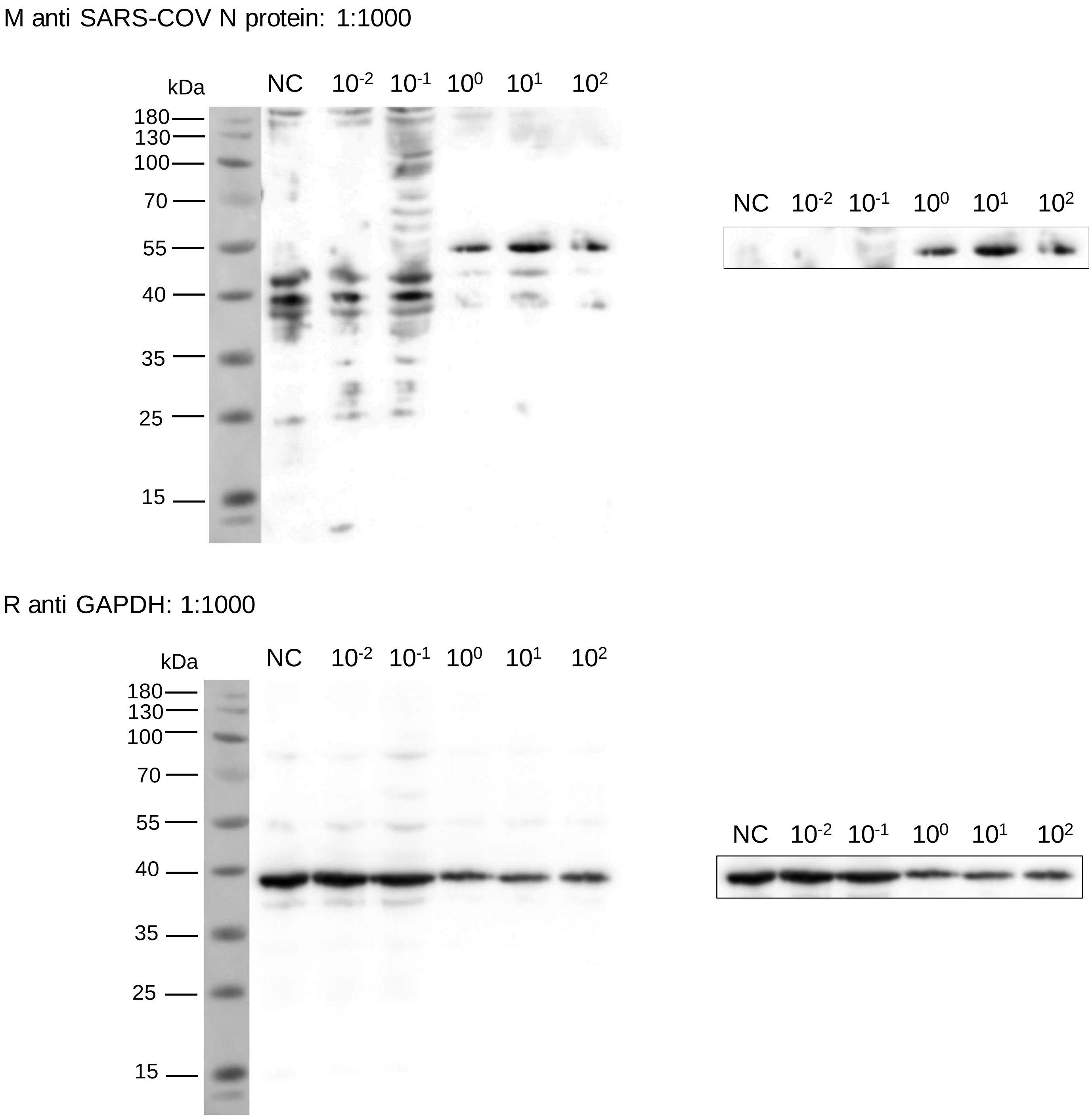

Figure S1. Western blots for SARS-CoV N protein and GAPDH control using SARS-CoV-2 (BEI_ USA-WA1/2020) cultivated in Vero E6 cells. Infections were initiated at titers of 10^{2} to 10^{-2} , and the rsulting lysates were analyzed by western blot using the same process as described for environmental samples. The N protein can be seen between 40 and 55 kD in the top panel.

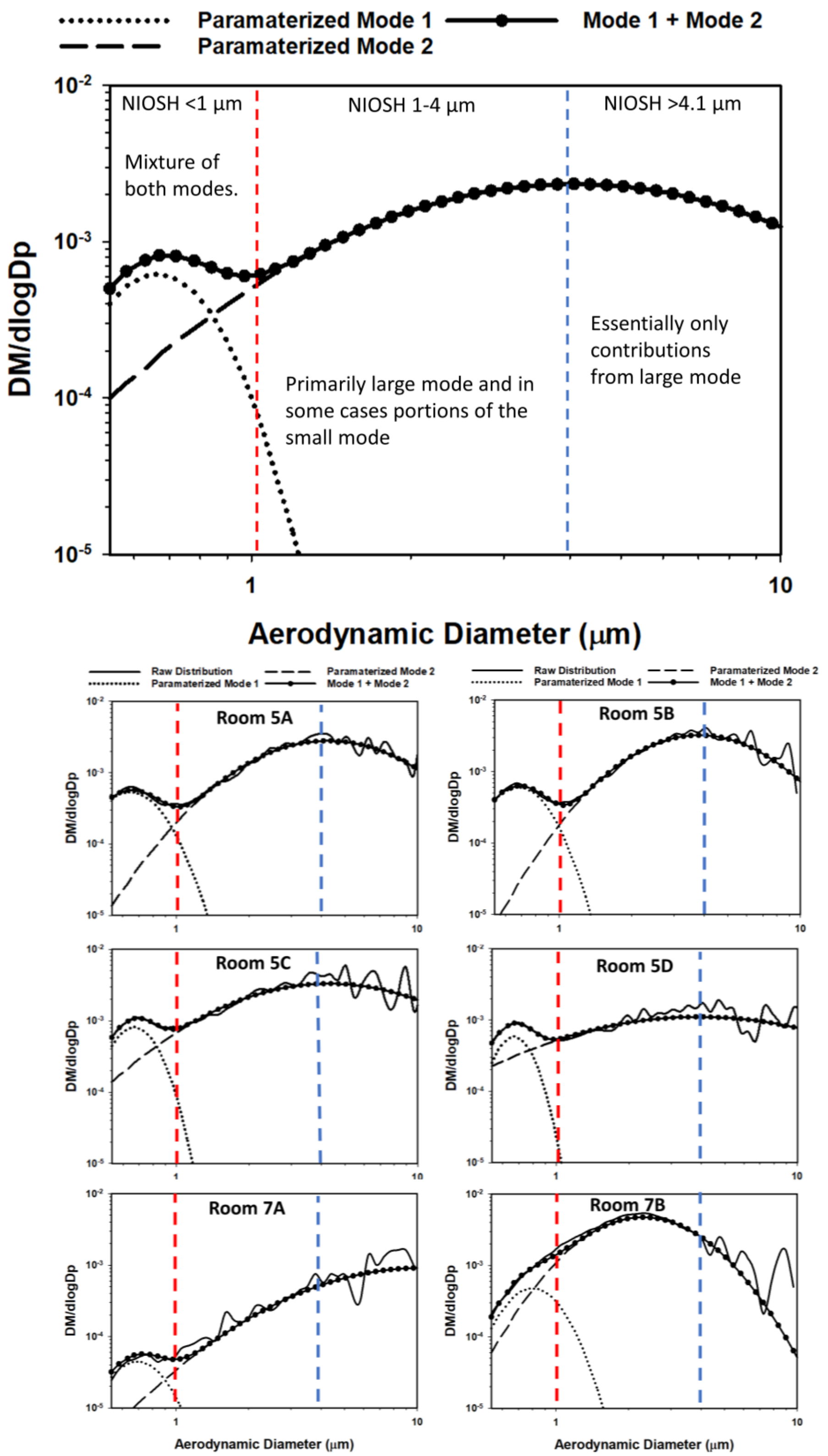

Figure S2. Aerosol Mass Distributions (assuming unit density particles), including measured (solid lines), the parameterized small mode (dots with no line), parameterized large mode (dashed line) and the combination of the two modes (heavy dots with line) for the average of all rooms in ward 5 (top), and each individual room in the study (bottom 6 panels). The red and blue lines indicate the cut-off size for the BC251 sampler, and indicate which fraction of each of the total aerosol and each mode was collected by the BC251 sampler.
